# Heavy Ion Radiation Directly Induced the Shift of Oral Microbiota and Increased the Cariogenicity of *Streptococcus mutans*

**DOI:** 10.1128/spectrum.01322-23

**Published:** 2023-06-13

**Authors:** Zheng Wang, Ge Yang, Xuedong Zhou, Xian Peng, Mingyun Li, Miaomiao Zhang, Dong Lu, Deqin Yang, Lei Cheng, Biao Ren

**Affiliations:** a State Key Laboratory of Oral Diseases & National Clinical Research Center for Oral Diseases & West China Hospital of Stomatology, Sichuan University, Chengdu, China; b Institute of Modern Physics, Chinese Academy of Sciences, Lanzhou, China; c College of Stomatology, Chongqing Medical University, Chongqing, China; The Ohio State University Division of Biosciences

**Keywords:** heavy ion, *Streptococcus mutans*, oral microbiota

## Abstract

Radiation caries is one of the most common complications of head and neck radiotherapy. A shift in the oral microbiota is the main factor of radiation caries. A new form of biosafe radiation, heavy ion radiation, is increasingly being applied in clinical treatment due to its superior depth-dose distribution and biological effects. However, how heavy ion radiation directly impacts the oral microbiota and the progress of radiation caries are unknown. Here, unstimulated saliva samples from both healthy and caries volunteers and caries-related bacteria were directly exposed to therapeutic doses of heavy ion radiation to determine the effects of radiation on oral microbiota composition and bacterial cariogenicity. Heavy ion radiation significantly decreased the richness and diversity of oral microbiota from both healthy and caries volunteers, and a higher percentage of Streptococcus was detected in radiation groups. In addition, heavy ion radiation significantly enhanced the cariogenicity of saliva-derived biofilms, including the ratios of the genus Streptococcus and biofilm formation. In the Streptococcus mutans-Streptococcus sanguinis dual-species biofilms, heavy ion radiation increased the ratio of S. mutans. Next, S. mutans was directly exposed to heavy ions, and the radiation significantly upregulated the *gtfC* and *gtfD* cariogenic virulence genes to enhance the biofilm formation and exopolysaccharides synthesis of S. mutans. Our study demonstrated, for the first time, that direct exposure to heavy ion radiation can disrupt the oral microbial diversity and balance of dual-species biofilms by increasing the virulence of S. mutans, increasing its cariogenicity, indicating a potential correlation between heavy ions and radiation caries.

**IMPORTANCE** The oral microbiome is crucial to understanding the pathogenesis of radiation caries. Although heavy ion radiation has been used to treat head and neck cancers in some proton therapy centers, its correlation with dental caries, especially its direct effects on the oral microbiome and cariogenic pathogens, has not been reported previously. Here, we showed that the heavy ion radiation directly shifted the oral microbiota from a balanced state to a caries-associated state by increasing the cariogenic virulence of S. mutans. Our study highlighted the direct effect of heavy ion radiation on oral microbiota and the cariogenicity of oral microbes for the first time.

## INTRODUCTION

Head and neck cancer (HNC) is the seventh most common malignancy worldwide. According to cancer statistics reports from 2023, HNC accounts for over 5% of all cancer new cases and over 2.8% of all cancer deaths in the United States (1). Radiotherapy, one of the integral treatment options for HNC, has significantly improved patient survival (2). However, ionizing radiation also damages normal tissues around tumors, which can lead to a series of acute and chronic oral complications during both intra-therapy and post-therapy stages (3). Radiation caries is a typical chronic complication in teeth which progresses rapidly and is extremely destructive. Currently, a number of factors are considered to be related to radiation caries, including radiation-induced hyposalivation, decreased dentin microhardness, increased carbohydrate intake, and a shift in oral microbiota towards cariogenic flora (4, 5), in which the composition and variation of oral microbial communities has been reported to be strongly correlated with radiation caries (6, 7).

Over the past few decades, photon beam (X-ray) has been the most common radiation type used in HNC radiotherapy (8). X-ray radiotherapy can damage the salivary gland, decreasing saliva flow and increasing the abundance of acidogenic and cariogenic microorganisms (9). Our previous studies showed for the first time that X-ray radiation induces a “silent mutation” in *gtfB*, the major cariogenic virulence gene of S. mutans, and reduced its cariogenic capabilities (10), indicating that radiotherapy can not only damage the host but might also directly affect the oral microbiota and the virulence of oral microbes. To minimize radiotherapy-related complications, clinically used radiation continues to evolve with the improvement of radiation technology (8). Heavy ion (generally carbon ion) radiotherapy is a cutting-edge oncology technique which has demonstrated greater local control of tumors and lower damage to healthy tissue in HNC patients (11, 12). The advantages of heavy ion therapy over X-ray radiotherapy are mainly due to its unique physical characteristics (13). Briefly, a heavy ion beam deposits energy across a very narrow range of depth, called the “Bragg peak,” onto a tumor, allowing reduced irradiation of normal tissues in the treatment field. Over the past decade, increased use of heavy ion radiotherapy in the United States, Europe, and Japan has produced excellent results in patients with HNCs such as mucosal malignant melanoma and adenoid cystic carcinoma (14). However, the relationship between heavy ion and radiation-related complications, especially radiation caries, remains unclear. Heavy ion radiation can induce DNA double-strand breakage, and cells are more prone to experience genetic mutations during DNA repair based on the homologous recombination and nonhomologous end-joining pathways than single-strand breaks caused by X-rays. Thus, heavy ion irradiation is frequently applied in mutation breeding to screen for promising strains or plant lines with application potential and research value (15, 16). In the development of radiation caries, heavy ion radiation may also directly induce genetic mutations in oral microbes, affecting the balance of the oral microbiota, but these effects are still unknown.

Therefore, in this study, to investigate the influence of heavy ion radiation on oral microbiota and their cariogenicity, saliva samples were directly exposed to heavy ions to analyze the microecological shift. The cariogenic virulence of dual-species and single-species biofilms of related bacteria was also compared before and after the heavy ion radiation to reveal the direct effects of radiation on cariogenic bacteria.

## RESULTS

### Heavy ion radiation decreased the richness and diversity of oral microbiota, but increased the abundance of the genus *Streptococcus*.

**FIG 1:**
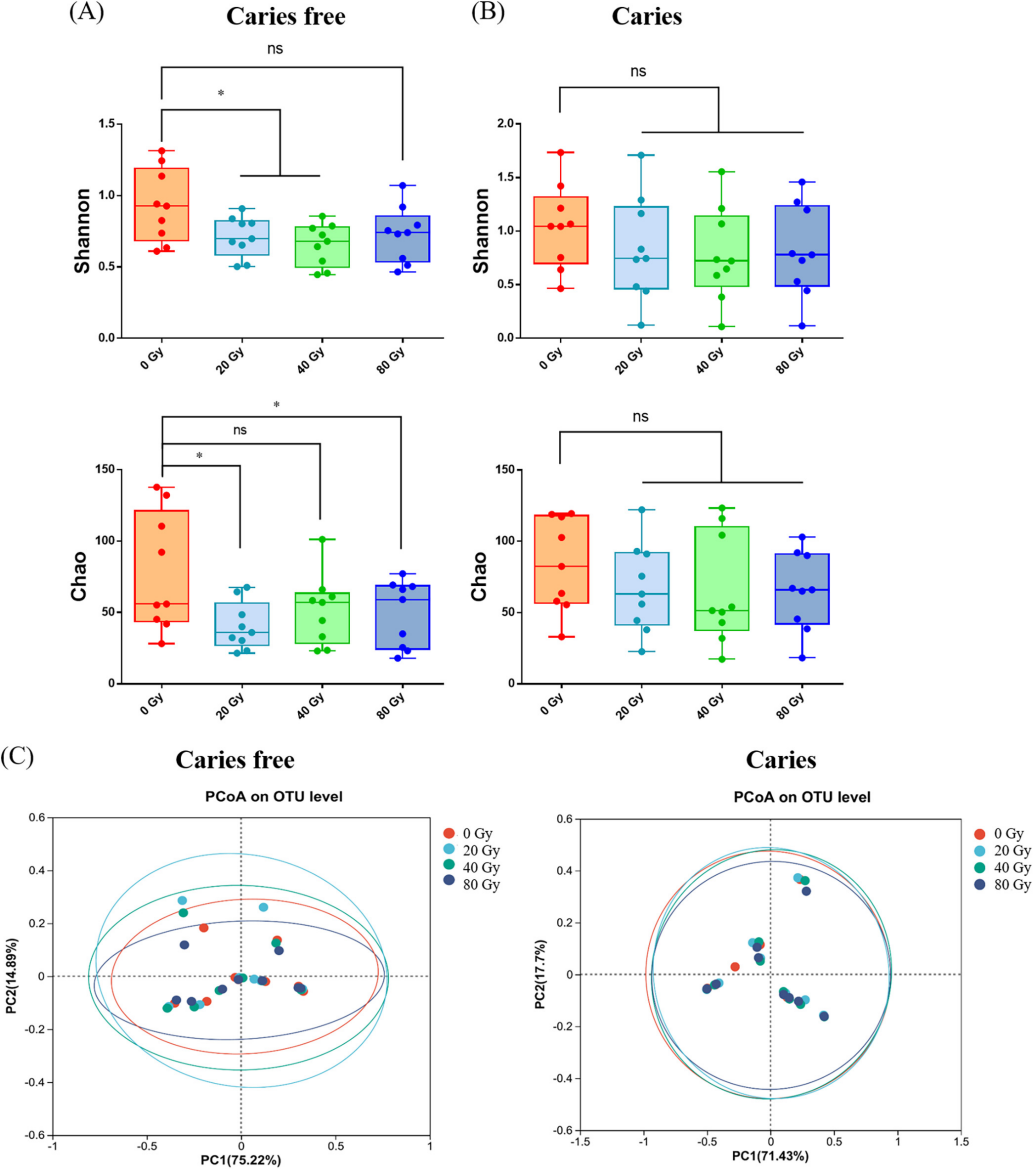
Heavy ion radiation decreases oral microbial richness and diversity. (A) Shannon and Chao1 indices of saliva-derived biofilms from caries-free donors (mean ± standard deviation [SD]; *n* = 9). (B) Shannon and Chao1 indices of saliva-derived biofilms from caries donors (mean ± SD; *n* = 9). (C) PCoA analysis of comparison between control group and radiation groups. ns, not significant; *, *P* < 0.05. OTU, operational taxonomic unit.

Saliva-derived biofilms from both caries-free and caries volunteers were directly exposed to heavy ion radiation at therapeutic dosages. As shown in Fig. 1A, for caries-free samples, the richness of the 20-Gy and 80-Gy groups was significantly lower than that of the control group (0 Gy), and the microbial diversity significantly decreased after radiation at 20-Gy and 40-Gy doses, indicating that heavy ion radiation can shift the balance of the healthy oral microbiota. However, for caries samples, although the Chao1 and Shannon indices decreased with increasing doses of radiation, there were no significant differences between the control and radiation groups (Fig. 1B). Similarly, principal-coordinate analysis (PCoA) showed that the radiation groups were quite different from the control group in caries-free samples, while the radiation groups showed no obvious differences from the control group in caries samples, indicating that the heavy ion radiation significantly affected the similarity of oral bacterial community structures and the microecology of caries free samples.

**FIG 2:**
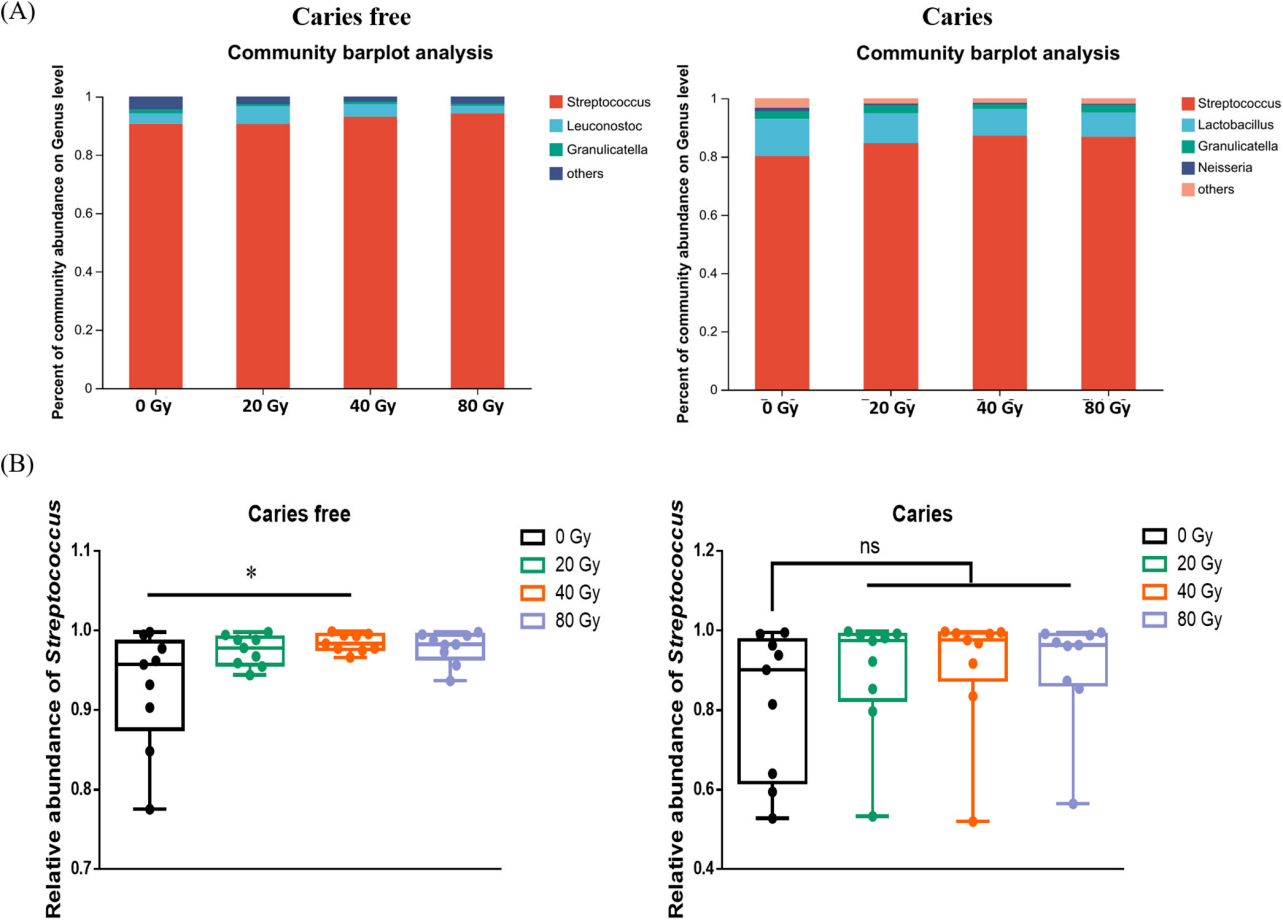
Heavy ion radiation disrupts microbial composition. (A) Abundance of microorganisms at the genus level in saliva-derived biofilms. (B) Compositional changes of Streptococcus between control group and radiation groups. ns, not significant; *, *P* < 0.05.

Next, a taxonomic composition analysis revealed that the major identified genera from caries-free samples were Streptococcus, *Leuconostoc*, and *Granulicatella*, while the dominant genera in caries samples included Streptococcus, *Lactobacillus*, *Granulicatella*, and *Neisseria* (Fig. 2A). Notably, in both caries-free and caries samples, Streptococcus had a strong correlation with radiation dosage, as Streptococcus abundance increased to its highest level under 80-Gy treatment. Specifically, the relative abundance of Streptococcus increased significantly in the 40-Gy group compared to the control group in caries-free samples (Fig. 2B), suggesting that the shift in oral microbiota could be due to the increase in Streptococcus induced by heavy ion radiation.

To confirm whether the increase in Streptococcus was only caused by heavy ion radiation and not by X-ray radiation, we then randomly isolated 3 samples from caries and caries-free donors, respectively, to explore the effect of X-ray radiation on oral microbiota. There was no significant difference in the Shannon and Chao1 indices between the radiation groups and the control group (Fig. S1A and B in the supplemental material). In addition, for caries-free samples, the relative abundance of Streptococcus was significantly decreased in the 20-Gy group, but recovered to a similar level compared to the control group, while Streptococcus remained at a stable level in all groups for caries samples (Fig. S1C), indicating that X-ray radiation could not increase the abundance of Streptococcus.

### Heavy ion radiation increased the ratio of *S. mutans* to *S. sanguinis* in saliva-derived biofilm.

**FIG 3:**
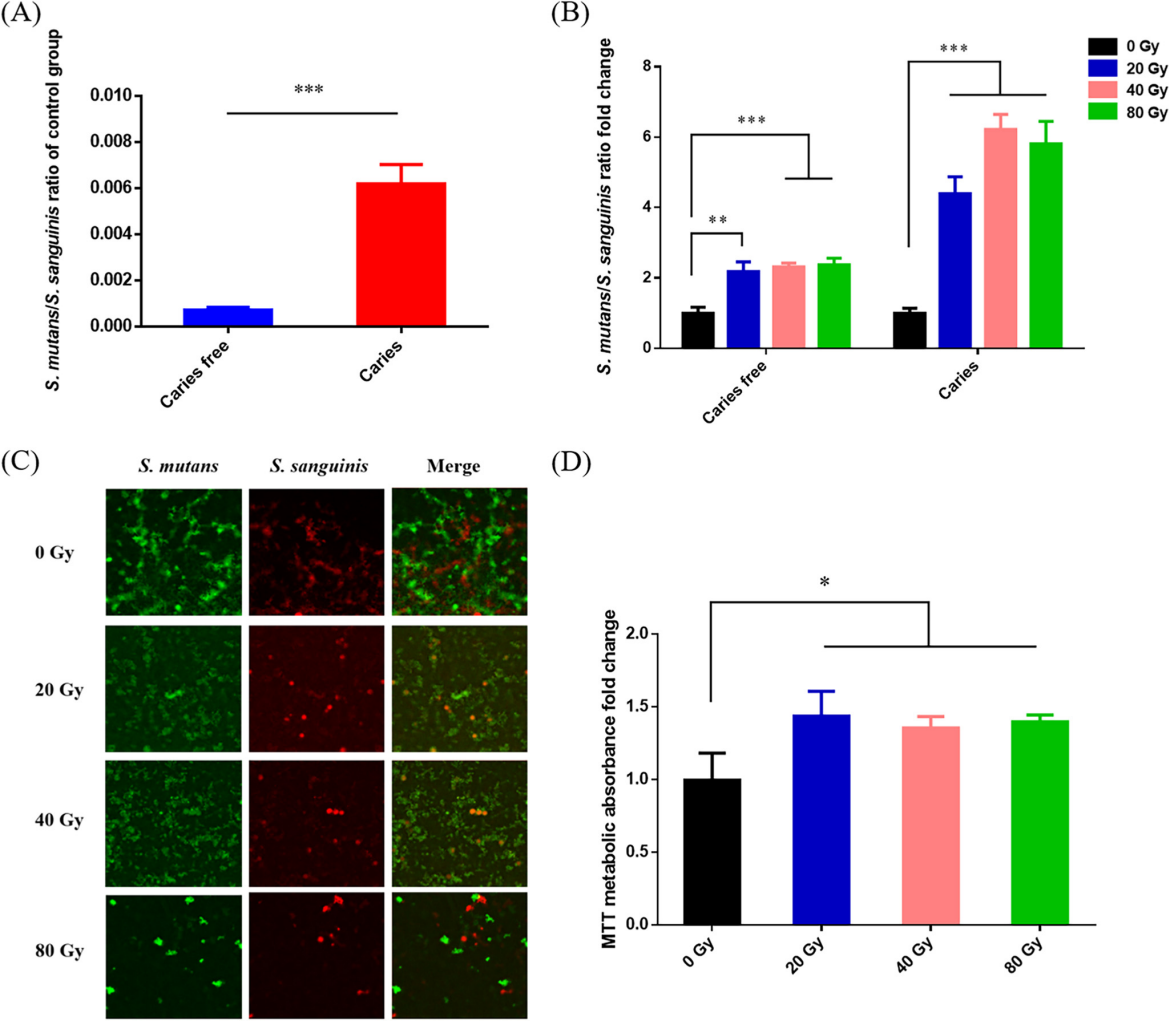
Heavy ion induces dual-species disturbance of saliva-derived biofilm and enhances its cariogenicity. (A) Ratio of S. mutans to S. sanguinis in saliva-derived biofilm of control group (0 Gy) subjected to qPCR (mean ± SD; *n* = 3). (B) Ratio fold-change of S. mutans to S. sanguinis in saliva-derived biofilm subjected to qPCR after radiation treatment (mean ± SD; *n* = 3). (C) FISH images of dual-species in mixed saliva-derived biofilm (S. mutans, stained green; S. sanguinis, stained red). (D) Results of MTT assay (mean ± SD; *n* = 3). *, *P* < 0.05; **, *P* < 0.01; ***, *P* < 0.001.

The ratio of S. mutans and S. sanguinis from the genus Streptococcus is highly related to the cariogenicity of oral microbiota. We first analyzed the levels of S. mutans and S. sanguinis in a saliva-derived biofilm of the control group (0 Gy) and found that the S. mutans/S. sanguinis ratio in the caries group was significantly higher than that in the caries free group (Fig. 3A), which is consistent with the caries degree trend of the volunteers. After heavy ion radiation, the S. mutans/S. sanguinis ratio significantly increased in both the caries and caries-free groups; in particular, the S. mutans/S. sanguinis ratio from the 40-Gy group was almost 6-fold greater than that of the control group in the caries samples (Fig. 3B). This result was further confirmed by fluorescent *in situ* hybridization in saliva-derived biofilms, as S. mutans showed a competitive advantage compared to S. sanguinis along with increasing radiation dosages (Fig. 3C). To further investigate whether the alteration of the S. mutans/S. sanguinis ratio induced by radiation affected the biofilm formation of oral flora, we performed an MTT [3-(4,5-dimethylthiazol-2-yl)-2,5-diphenyltetrazolium bromide] assay. As shown in Fig. 3D, the heavy ion radiation significantly enhanced biofilm formation compared to that in the control group, suggesting that an increase in the S. mutans/S. sanguinis ratio could promote biofilm formation in saliva-derived biofilm.

### Heavy ion radiation promoted the ratio of *S. mutans* within the *S. mutans*-*S. sanguinis* dual-species biofilm.

**FIG 4:**
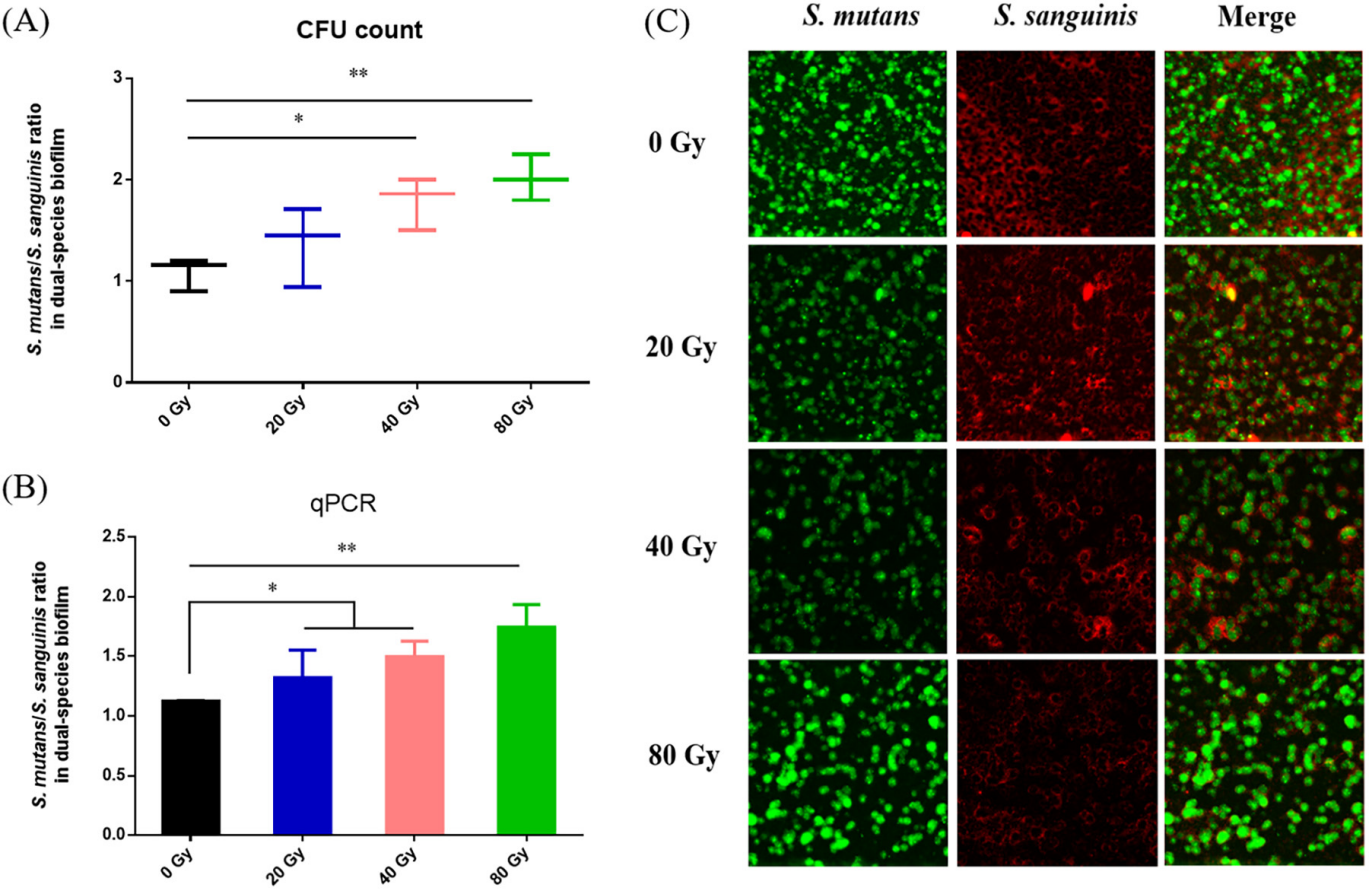
The ratio of S. mutans within dual-species biofilm increased after radiation treatment. (A) Ratio of S. mutans to S. sanguinis in dual-species biofilm by CFU count (mean ± SD; *n* = 3). (B) Ratio of S. mutans to S. sanguinis in dual-species biofilm by qPCR (mean ± SD; *n* = 3). (C) FISH images of dual-species biofilm (S. mutans, stained green; S. sanguinis, stained red). *, *P* < 0.05; **, *P* < 0.01.

Because heavy ion radiation enhanced the S. mutans/S. sanguinis ratio in saliva-derived biofilms, we then constructed a S. mutans-S. sanguinis dual-species biofilm. Following heavy ion radiation, the proportion of S. mutans increased significantly in the S. mutans-S. sanguinis dual-species biofilms; in particular, the proportion of S. mutans in the 80-Gy group was approximately 2/3 within the dual-species biofilm, remarkably higher than that of S. sanguinis (Fig. 4A and B). The FISH (fluorescent *in situ* hybridization) staining results also showed that the heavy ion radiation significantly increased the ratio of S. mutans within the dual-species biofilms; in particular, there was less S. sanguinis in the 80-Gy group (Fig. 4C), indicating that heavy ion radiation could increase the ratio of S. mutans to cause imbalance in dual-species biofilms.

### Heavy ion radiation promoted the biofilm formation and exopolysaccharides synthesis of *S. mutans*.

**FIG 5:**
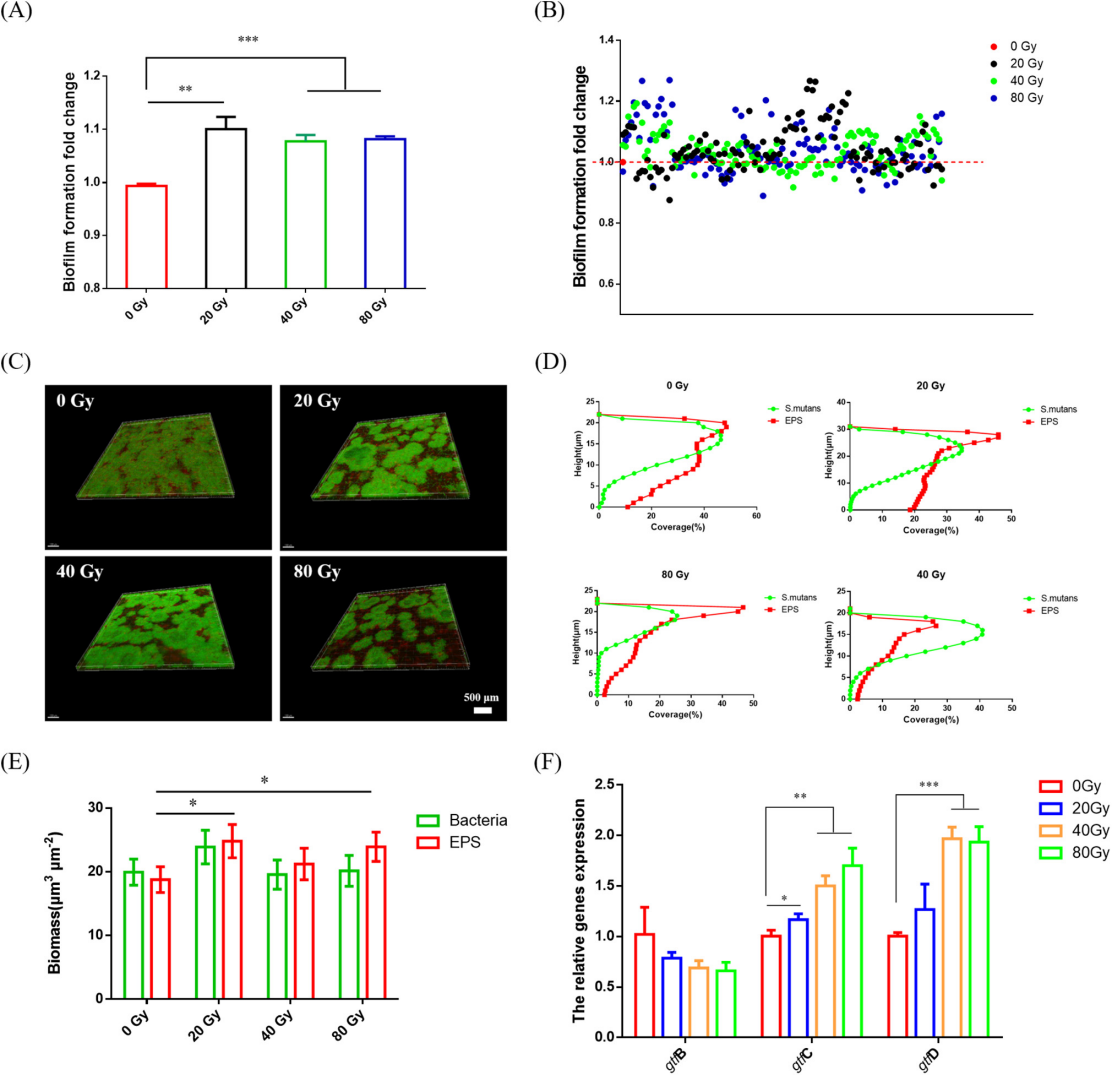
Heavy ion radiation promotes biofilm formation and exopolysaccharide synthesis in S. mutans. (A) Biofilms formation fold-change of S. mutans in radiation groups based on crystal violet assay (mean ± SD; *n* = 3). (B) Biofilm formation fold-change of different S. mutans isolates (mean ± SD; *n* = 3). (C) Three-dimensional reconstruction of S. mutans biofilms (bacteria stained green, EPS stained red). (D) Distributions of bacteria and EPS coverage (%) at different heights (given in μm). (E) Biomasses of EPS and bacteria (μm^3^ μm^−2^) calculated according to 3 random views of S. mutans biofilms (mean ± SD; *n* = 3). (F) Expression of EPS synthesis-related genes of S. mutans (mean ± SD; *n* = 3). *, *P* < 0.05; **, *P* < 0.01; ***, *P* < 0.001.

Due to the strong effects of heavy ion radiation on the S. mutans in the S. mutans-S. sanguinis dual-species biofilms, we then directly investigated the impact of heavy ion radiation on S. mutans single-species biofilms. As shown in Fig. 5A, heavy ion radiation significantly increased the biofilm formation of S. mutans compared to that in the control groups. We then further isolated 351 S. mutans single colonies from the radiation groups and found that 74 strains exhibited stronger biofilm formation than that of the control group, while 11 strains exhibited reduced ability (Fig. 5B).

Because the S. mutans cells from radiation groups showed enhanced biofilm formation, we then measured the major cariogenic virulence factor water-insoluble extracellular polysaccharides (EPS). As shown in Fig. 5C and D, bacteria (green) and EPS (red) were uniformly distributed from top to bottom in the control group, while most of the EPS of the 20-Gy and 80-Gy groups were distributed at the top. Biomass calculation indicated that there was no significant difference in the total number of bacteria under different radiation conditions; however, much more EPS was produced in the biofilms from the 20-Gy and 80-Gy groups compared to control group (Fig. 5E), indicating that heavy ion radiation increase the EPS production to promote the biofilm formation of S. mutans. The mRNA expression levels of glucosyltransferase-related genes were then measured. Although the expression of *gtfB* showed no difference, *gtfC* and *gtfD* mRNA expression was significantly upregulated (Fig. 5F), consistent with the increased EPS production.

## DISCUSSION

It is well known that a change in oral microbiota is considered one of the major risk factors for oral complications following radiotherapy treatment. The direct and indirect effects of ionizing radiation are responsible for the microbial changes, such as decreased salivary function, different dietary habits, and radiation-induced virulence variation (17, 18). Here, to investigate the direct effect of heavy ion radiation on oral microbiota, saliva samples and caries-related pathogens received therapeutic doses of radiation *in vitro*, eliminating adverse effects caused by individual differences. We found that the heavy ion radiation directly shifted the oral microbiota from a balanced state to a caries-associated state by increasing the cariogenic virulence of S. mutans.

With the development of pyrosequencing, a large number of microorganisms that could not be identified by traditional culture methods have been revealed to be associated with dental caries, and microbiome dysbiosis is considered one of the main factors leading to caries (19, 20). Previous studies have demonstrated a negative correlation between microbial diversity and caries severity (21). Thus, radiation caries was suggested to be caused by radiation-induced hyposalivation, which leads to an overgrowth of acidogenic species and a reduction in oral microorganism community diversity (9, 22). Interestingly, our results revealed that oral flora diversity declined after direct exposure to heavy ion radiation, especially in the samples from caries-free donors. These results indicated that the direct effect of radiation was also a factor affecting the diversity of oral microbiota. Many studies have indicated that Streptococcus is a major contributor to caries progression because they can form biofilms and produce acid (23, 24), while in our study, the most obvious change was the increase in Streptococcus abundance. Moreover, it is also noteworthy that in the caries-free samples, *Leuconostoc* significantly reduced when the radiation dose reached 80 Gy in our study. Previous studies have revealed that Leuconostoc mesenteroides shows oral probiotic characteristics and possesses strong antagonistic activity against S. mutans (25), indicating that heavy ion radiation induced an increase in cariogenic Streptococcus and also reduced their antagonistic bacteria to shift the oral microflora.

Both S. mutans and S. sanguinis are representative oral bacteria during early plaque formation, and strongly associated with dental caries. Unlike S. mutans, S. sanguinis is considered a model commensal bacterium related to the establishment of healthy dental biofilms. H_2_O_2_ produced by S. sanguinis has been shown to antagonize colonization by S. mutans (26). Any factors which can alter the competition between S. mutans and S. sanguinis will influence biofilm conditions between a cariogenic or healthy state (27). In our study, heavy ion radiation significantly increased the S. mutans/S. sanguinis ratios both in saliva-derived biofilms and in their dual-species biofilms, indicating that the heavy ion radiation shifted the oral microbiota to a cariogenic state.

In the present study, the survival rates of S. mutans cells were 82%, 70%, and 45% in the 20-Gy, 40-Gy, and 80-Gy groups, respectively, all of which were lower than the survival rate of those that received X-ray radiation in our previous study (10). These results were consistent with another study in which carbon ion beam radiation had a markedly more damaging effect on Saccharomyces cerevisiae cells compared to X-ray radiation (28). These differences may result from the high-linear energy transfer form of heavy ion radiation, which causes clustered damage to induce more serious DNA double-strand breaks (29).

It is known that S. mutans can increase its attachment to teeth and its cariogenic ability by synthesizing extra- and intracellular polysaccharides regulated by three glucosyltransferases (Gtfs). Among these, GtfB is responsible for synthesizing water-insoluble glucans, GtfC mediates the production of a mixture of water-soluble and -insoluble glucans, and GtfD synthesizes water-soluble glucans (30). Our results showed that S. mutans had higher biofilm formation and EPS production following radiation, while *gtfC* and *gtfD* were significantly upregulated in the radiation groups compared to the control group, indicating that heavy ion radiation increased the cariogenic virulence of S. mutans. Further investigations should focus on virulence variation at the genetic level to identify the direct effects of heavy ion radiation on the genomes of different cariogenic bacterial isolates.

**FIG 6:**
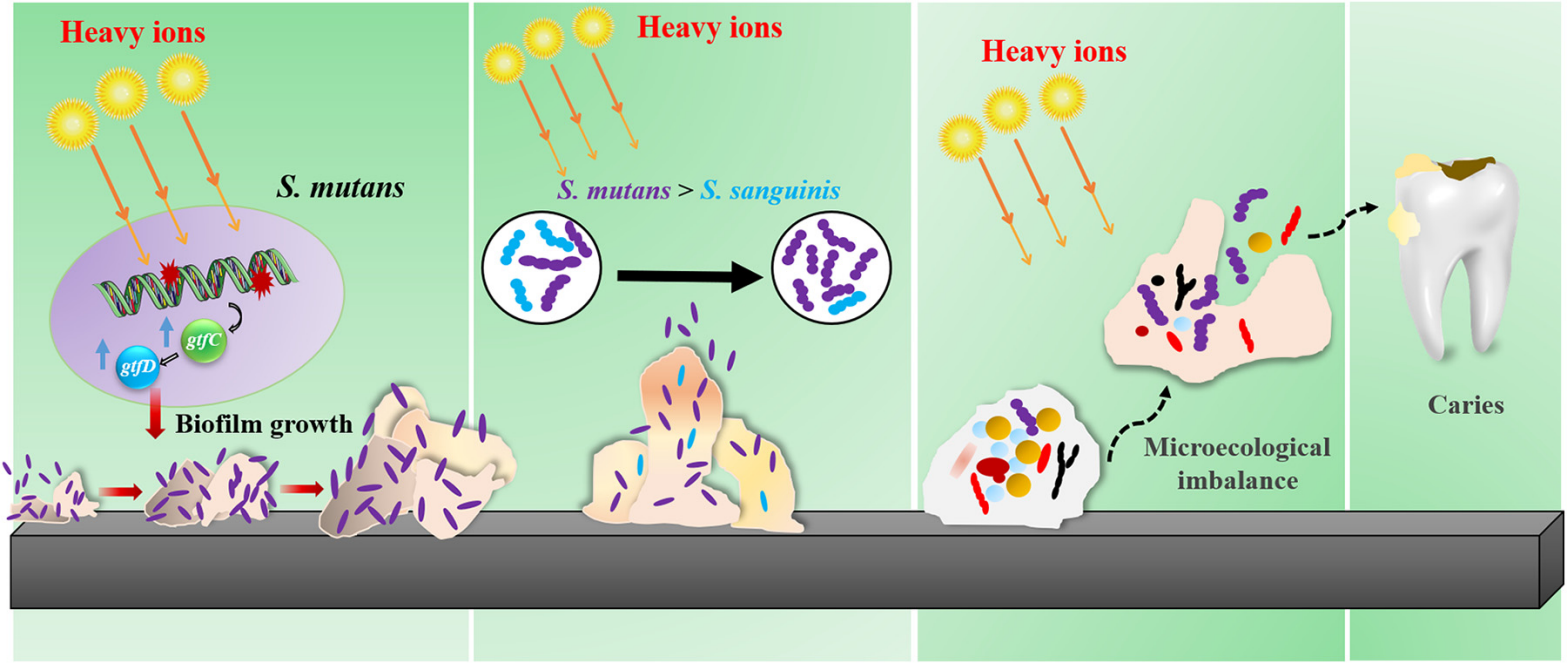
Working hypothesis for the shift in oral microbiota and increased cariogenicity of S. mutans induced by heavy ion radiation. Exposure to therapeutic doses of heavy ion radiation activates transcription of glucosyltransferase-related genes, thereby triggering biofilm formation and exopolysaccharide synthesis in S. mutans, which may consequently lead to a competitive advantage over S. sanguinis within a dual-species biofilm. These alterations to the composition of potential caries-associated species may further decrease the diversity of oral microbiota and undermine the balance of the oral microflora system, causing the microbial population to shift toward cariogenic flora.

In conclusion, we investigated the direct effect of high-energy heavy ion radiation on oral microbiota and cariogenic agents for the first time and provided potential information for the prevention and management of heavy ion radiation-induced oral complications. After exposure to therapeutic doses of heavy ion radiation, the oral microecology exhibited decreased diversity and developed a potential caries-associated species composition. The radiation enhanced biofilm formation and exopolysaccharide synthesis in S. mutans and increased the S. mutans/S. sanguinis ratio in both saliva-derived biofilms and dual-species biofilms (Fig. 6).

## MATERIALS AND METHODS

### Saliva collection and bacterial culture.

In the present study, saliva collection was approved by the Ethical Committee of West China Hospital of Stomatology, Sichuan University (Chengdu, China) (project identification code: WCHSIRB-D-2016-182; approval date: 25 December 2016). Unstimulated saliva was collected from 9 caries (decayed, missing and filled teeth [DMFT] ≥ 1) and 9 caries-free (DMFT = 0) adult donors. None of the donors had systemic diseases or had received antibiotics within 3 months prior to this study. All participants were ordered not to brush their teeth for 24 h before saliva collection and fasted for at least 2 h prior to donating saliva. Saliva samples were collected in 50-mL sterile centrifuge tubes and immediately transported on ice to the laboratory. Next, saliva samples were centrifuged at 2,000 × *g* for 10 min to remove food debris and eukaryotic cells, and each sample was diluted in sterile glycerol to a saliva concentration of 70% and stored at −80°C for further radiation (31).

S. mutans UA159 and S. sanguinis SK1 (ATCC 10556) were obtained from the State Key Laboratory of Oral Diseases (Sichuan University, Chengdu, China). All bacteria were routinely grown anaerobically at 37°C (90% N_2_, 5% CO_2_, 5% H_2_) in brain heart infusion broth (BHI; Difco, Sparks, MD). For dual-species samples, S. mutans and S. sanguinis cells were harvested at the mid-logarithmic phase by centrifugation (4,000 × *g*, 4°C, 10 min), washed twice with phosphate-buffered saline (PBS), and re-suspended (optical density at 600 nm [OD_600_] = 0.5) in the same solution, and bacterial suspensions were mixed at a 1:1 ratio. For single-species samples, S. mutans were harvested at the mid-logarithmic phase, washed with PBS, and re-suspended in PBS (OD_600_ = 0.5) (32).

### Heavy ion irradiation treatment.

Irradiation treatments were performed at the Heavy Ion Research Facility in Lanzhou (HIRFL), which can generate radiation beams at different intensities by accelerating heavy ions (33). We used carbon ion C^6+^ beams in this study and designed four irradiation groups: 0, 20, 40, and 80 Gy. Next, 2-mL volumes of saliva or bacterial samples were transferred to 35-mm tissue culture plates sealed with parafilm and stably irradiated, while the control group (0 Gy) was subjected to the same handling minus irradiation. After radiation treatment, all samples were immediately diluted in sterile glycerol and stored at −80°C for further experiments.

### Saliva-derived biofilm formation and 16S rRNA sequencing.

In this study, saliva-derived biofilm was cultured using SHI medium with components described in a previous report (34). Next, 200 μL of each irradiated saliva samples was inoculated into 24-well plates containing 1.8 mL SHI medium. Then plates were incubated at 37°C under anaerobic conditions. After 24 h of biofilm formation, the total DNA of saliva-derived biofilms samples was extracted, amplified, and sequenced by Shanghai Majorbio Bio-pharm Technology Co., Ltd. (Shanghai, China) according to their standard procedures (supplemental material).

### Viable population determined by plate-count method.

For CFU counts of dual-species samples, the irradiated dual-species samples were diluted and spread on solid BHI medium to determine the counts of viable S. mutans and S. sanguinis after 48 h of incubation at 37°C (90% N_2_, 5% CO_2_, 5% H_2_).

### DNA isolation and real-time PCR.

To determine the ratio of S. mutans to S. sanguinis in saliva-derived biofilm, 200 μL of irradiated saliva samples was inoculated into 24-well plates containing 1.8 mL SHI medium and incubated at 37°C under anaerobic conditions. After 24 h of biofilm formation, the total DNA of saliva-derived biofilms samples was extracted by a TIANamp Bacteria DNA kit (TIANGEN, Beijing, China) according to the manufacturer’s instructions. DNA purity and concentration tests were performed using a NanoDrop 2000 spectrophotometer (Thermo Fisher Scientific, Waltham, MA, USA). The amount of DNA was determined by quantitative real-time PCR on a C1000 Touch Thermal Cycler instrument (Bio-Rad, Philadelphia, PA, USA), and reflects the number of S. mutans and S. sanguinis (35). The sequences of the primers for the dual-species samples are listed in Table 1.

**TABLE 1:** Specific primers used for quantification of dual-species samples

Bacterium	Direction	Template strand sequence (5′–3′)
S. mutans	F	GCCTACAGCTCAGAGATGCTATTCT
	R	GCCATACACCACTCATGAATTGA
S. sanguinis	F	GAGCGGATGGCCAATTATATCT
	R	CCGGATGATGTCGGCAATA

To determine the ratio of S. mutans to S. sanguinis in dual-species biofilm, irradiated dual-species suspensions were diluted at a concentration of 10^6^ CFU/mL and inoculated in 1 mL BHI with 1% sucrose in 48-well plates (37°C, 90% N_2_, 5% CO_2_, 5% H_2_). After 24 h of incubation, the supernatant was removed, and the biofilms were harvested. Then, DNA extraction and real-time PCR were performed as described above to calculate the number of S. mutans and S. sanguinis.

### Fluorescent *in situ* hybridization.

FISH was used to reflect the distribution and composition of S. mutans and S. sanguinis in saliva and dual-species biofilms after irradiation. Saliva and dual-species biofilms were cultured as described above, and the saliva sample was mixed in advance by all collected saliva samples from caries and caries-free donors. Biofilms established at 24 h were fixed in 4% paraformaldehyde (PFA) and bacteria were labeled with oligonucleotide probes (Table 2) and investigated by species-specific fluorescence in FISH as described previously (36). The biofilms were imaged using a confocal laser scanning microscope (Leica, Wetzlar, Germany) and micrographs were sampled from at least three randomly selected positions of each sample.

**TABLE 2:** Oligonucleotide probes used in fluorescent *in situ* hybridization

Designation	Nucleotide sequence (5′–3′)	Target
S. mutans	(Alexa Fluor 488)-ACTCCAGACTTTCCTGAC	S. mutans 16S rRNA
S. sanguinis	(Alexa Fluor 594)-GCATACTATGGTTAAGCCACAGCC	S. sanguinis 16S rRNA

### MTT assay.

The MTT assay is one of the most versatile and popular tools for estimating the metabolic activity of living cells (37). In the present study, an MTT assay was used to investigate the metabolic activity of saliva-derived biofilm. Briefly, 200 μL of irradiated saliva samples and 1.8 mL SHI medium was inoculated into 24-well plates containing a sterile saliva-coated hydroxyapatite (HA) tablet. After biofilm formation for 48 h at 37°C under anaerobic conditions (medium refreshed every 12 h), the HA tablet was washed with PBS to remove plankton biofilm from the sample surface. The HA tablet was then transferred to a new 24-well plate and inoculated with 1 mL MTT dye (0.5 mg/mL) at 37°C under 5% CO_2_ and light-tight conditions for 1 h. The HA tablet was then transferred to a new 24-well plate and inoculated with 1 mL of dimethyl sulfoxide (DMSO) for 20 min with gentle mixing at room temperature. Next, 200 μL of the DMSO solution from each well was transferred to a new 96-well plate, and the absorbance was measured at 540 nm using a Thermo Scientific Multiskan GO reader (Thermo Fisher Scientific, Waltham, MA).

### The crystal violet assay.

Irradiated S. mutans suspensions were diluted at a concentration of 10^6^ CFU/mL and inoculated in 2 mL BHI with 1% sucrose in 24-well plates (37°C, 90% N_2_, 5% CO_2_, 5% H_2_) containing sterile slides. After 24 h of biofilm formation, the supernatant was removed and the biofilms were washed twice with PBS, fixed with methanol for 15 min, and stained with 0.1% crystal violet solution for 5 min. The crystal violet-stained biofilms were then solubilized in 95% ethanol with shaking and 200 μL of ethanol was transferred to a new 96-well plate. The absorbance of the solution was measured by a Thermo Scientific Multiskan GO reader (Thermo Fisher Scientific) at 595 nm (38).

For single-colony screening, cell suspensions from irradiation groups were diluted, spread on solid BHI medium, and incubated at 37°C for 48 h. Crystal violet assays were then performed with the isolated strains.

### Extracellular polysaccharide staining.

S. mutans biofilms were cultured as described in the crystal violet assay. EPS was labeled with 2.5 μM Alexa Fluor 647-labeled dextran conjugate (Molecular Probes, Eugene, OR) at the beginning of biofilm formation, and the bacteria were stained with 2.5 μM SYTO9 (Molecular Probes) at 15 min after biofilm formation. Biofilm images were captured with a Leica DMIRE2 confocal laser scanning microscope (Leica, Wetzlar, Germany) equipped with a ×60 oil immersion lens objective. Three-dimensional reconstruction of biofilms was performed with IMARIS version 7.0.0 (Bitplane, Zurich, Switzerland). The biomasses of EPS and bacterial cells were calculated with COMSTAT image-processing software according to the fluorescence value (39).

### RNA extraction and quantitative reverse transcriptase PCR.

Total RNA of S. mutans biofilms was extracted and purified as previously described (40). cDNA reverse transcription was then performed using the cDNA synthesis kit (TaKaRa, Shiga, Japan). Reverse transcriptase quantitative PCR (qRT-PCR) was performed using TB green Premix *Ex Taq* (TaKaRa) on a C1000 Touch Thermal Cycler instrument (Bio-Rad). The expression of *gtfB*, *gtfC*, and *gtfD* were analyzed according to the threshold cycle (2^–ΔΔ^*^CT^*)method. The primers used in this section are shown in Table 3.

**TABLE 3:** Specific primers used for qPCR

Gene	Direction	Template strand sequence (5′–3′)
16s RNA	F	AGCGTTGTCCGGATTTATTG
	R	CTACGCATTTCACCGCTACA
*gtfB*	F	CACTATCGGCGGTTACGAAT
	R	CAATTTGGAGCAAGTCAGCA
*gtfC*	F	GATGCTGCAAACTTCGAACA
	R	TATTGACGCTGCGTTTCTTG
*gtfD*	F	TTGACGGTGTTCGTGTTGAT
	R	AAAGCGATAGGCGCAGTTTA

### Statistical analysis.

In the present study, nonparametric Mann–Whitney analyses were used to compare the alpha diversity and abundance of microorganisms, while one-way analyses of variance and Student’s *t* tests were used for other *in vitro* studies. All experiments were repeated at least 3 times independently. Differences were considered significant when *P* < 0.05. Statistical analysis was performed with SPSS software version 16.0 (SPSS Inc., Chicago, IL, USA).

### Data availability.

 Raw read files have been deposited in the NCBI Sequence Read Archive (SRA) database (accession no. PRJNA931456).

## References

[1] Siegel RL, Miller KD, Wagle NS, Jemal A. 2023. Cancer statistics, 2023. CA Cancer J Clin 73:17–48. doi:10.3322/caac.21763. 36633525

[2] Alterio D, Marvaso G, Ferrari A, Volpe S, Orecchia R, Jereczek-Fossa BA. 2019. Modern radiotherapy for head and neck cancer. Semin Oncol 46:233–245. doi:10.1053/j.seminoncol.2019.07.002. 31378376

[3] Sciubba JJ, Goldenberg D. 2006. Oral complications of radiotherapy. Lancet Oncol 7:175–183. doi:10.1016/S1470-2045(06)70580-0. 16455482

[4] Tsutsumi R, Goda M, Fujimoto C, Kanno K, Nobe M, Kitamura Y, Abe K, Kawai M, Matsumoto H, Sakai T, Takeda N. 2016. Effects of chemotherapy on gene expression of lingual taste receptors in patients with head and neck cancer. Laryngoscope 126:E103–E109. doi:10.1002/lary.25679. 26422579

[5] Lopes CCA, Rodrigues RB, Cenci MS, Uehara JLS, Maske TT, Limirio P, Soares PBF, Novais VR. 2021. Effect of ionizing radiation and cariogenic biofilm challenge on root-dentin caries. Clin Oral Invest 25:4059–4068. doi:10.1007/s00784-020-03736-0. 33765193

[6] Hu YJ, Shao ZY, Wang Q, Jiang YT, Ma R, Tang ZS, Liu Z, Liang JP, Huang ZW. 2013. Exploring the dynamic core microbiome of plaque microbiota during head-and-neck radiotherapy using pyrosequencing. PLoS One 8:e56343. doi:10.1371/journal.pone.0056343. 23437114 PMC3578878

[7] Gao L, Hu Y, Wang Y, Jiang W, He Z, Zhu C, Ma R, Huang Z. 2015. Exploring the variation of oral microbiota in supragingival plaque during and after head-and-neck radiotherapy using pyrosequencing. Arch Oral Biol 60:1222–1230. doi:10.1016/j.archoralbio.2015.05.006. 26073028

[8] Kim JK, Leeman JE, Riaz N, McBride S, Tsai CJ, Lee NY. 2018. Proton therapy for head and neck cancer. Curr Treat Options Oncol 19:28. doi:10.1007/s11864-018-0546-9. 29744681

[9] Aguiar GP, Jham BC, Magalhães CS, Sensi LG, Freire AR. 2009. A review of the biological and clinical aspects of radiation caries. J Contemp Dent Pract 10:83–89. doi:10.5005/jcdp-10-4-83. 19575058

[10] Wang Z, Zhou Y, Han Q, Ye X, Chen Y, Sun Y, Liu Y, Zou J, Qi G, Zhou X, Cheng L, Ren B. 2021. Synonymous point mutation of *gtfB* gene caused by therapeutic X-rays exposure reduced the biofilm formation and cariogenic abilities of *Streptococcus mutans*. Cell Biosci 11:91. doi:10.1186/s13578-021-00608-2. 34001238 PMC8130306

[11] Tsujii H, Kamada T. 2012. A review of update clinical results of carbon ion radiotherapy. Jpn J Clin Oncol 42:670–685. doi:10.1093/jjco/hys104. 22798685 PMC3405871

[12] Schulz-Ertner D, Nikoghosyan A, Thilmann C, Haberer T, Jäkel O, Karger C, Kraft G, Wannenmacher M, Debus J. 2004. Results of carbon ion radiotherapy in 152 patients. Int J Radiat Oncol Biol Phys 58:631–640. doi:10.1016/j.ijrobp.2003.09.041. 14751537

[13] Li X, Lee A, Cohen MA, Sherman EJ, Lee NY. 2020. Past, present and future of proton therapy for head and neck cancer. Oral Oncol 110:104879. doi:10.1016/j.oraloncology.2020.104879. 32650256

[14] Kamada T, Tsujii H, Blakely EA, Debus J, De Neve W, Durante M, Jäkel O, Mayer R, Orecchia R, Pötter R, Vatnitsky S, Chu WT. 2015. Carbon ion radiotherapy in Japan: an assessment of 20 years of clinical experience. Lancet Oncol 16:e93–e100. doi:10.1016/S1470-2045(14)70412-7. 25638685

[15] Guo X, Zhang M, Gao Y, Cao G, Yang Y, Lu D, Li W. 2019. A genome-wide view of mutations in respiration-deficient mutants of *Saccharomyces cerevisiae* selected following carbon ion beam irradiation. Appl Microbiol Biotechnol 103:1851–1864. doi:10.1007/s00253-019-09626-0. 30661110

[16] Zhang M, Cao G, Guo X, Gao Y, Li W, Lu D. 2018. A comet assay for DNA damage and repair after exposure to carbon-ion beams or X-rays in *Saccharomyces cerevisiae*. Dose Response 16:1559325818792467. doi:10.1177/1559325818792467. 30116170 PMC6088507

[17] Alamri OD, Cundy AB, Di Y, Jha AN, Rotchell JM. 2012. Ionizing radiation-induced DNA damage response identified in marine mussels, *Mytilus* sp. Environ Pollut 168:107–112. doi:10.1016/j.envpol.2012.04.015. 22609861

[18] Epstein JB, Thariat J, Bensadoun RJ, Barasch A, Murphy BA, Kolnick L, Popplewell L, Maghami E. 2012. Oral complications of cancer and cancer therapy: from cancer treatment to survivorship. CA Cancer J Clin 62:400–422. doi:10.3322/caac.21157. 22972543

[19] Chen T, Shi Y, Wang X, Wang X, Meng F, Yang S, Yang J, Xin H. 2017. High-throughput sequencing analyses of oral microbial diversity in healthy people and patients with dental caries and periodontal disease. Mol Med Rep 16:127–132. doi:10.3892/mmr.2017.6593. 28534987 PMC5482155

[20] Chen H, Jiang W. 2014. Application of high-throughput sequencing in understanding human oral microbiome related with health and disease. Front Microbiol 5:508. doi:10.3389/fmicb.2014.00508. 25352835 PMC4195358

[21] Xiao C, Ran S, Huang Z, Liang J. 2016. Bacterial diversity and community structure of supragingival plaques in adults with dental health or caries revealed by 16S pyrosequencing. Front Microbiol 7:1145. doi:10.3389/fmicb.2016.01145. 27499752 PMC4956651

[22] Dobroś K, Hajto-Bryk J, Wróblewska M, Zarzecka J. 2016. Radiation-induced caries as the late effect of radiation therapy in the head and neck region. Contemp Oncol (Pozn) 20:287–290. doi:10.5114/wo.2015.54081. 27688724 PMC5032152

[23] Lemos JA, Palmer SR, Zeng L, Wen ZT, Kajfasz JK, Freires IA, Abranches J, Brady LJ. 2019. The biology of *Streptococcus mutans*. Microbiol Spectr 7. doi:10.1128/microbiolspec.GPP3-0051-2018. PMC6615571 30657107

[24] Zhang Z, Ji Y, Liu D, Zhou S, Wang Z, Chen R, Li T, Zhao B, Yao H, Du M. 2023. Heat shock protein inhibitors show synergistic antibacterial effects with photodynamic therapy on caries-related streptococci *in vitro* and *in vivo*. mSphere 8:e00679-22. doi:10.1128/msphere.00679-22. 36853046 PMC10117063

[25] Gu M, Nguyen HT, Cho JH, Suh JW, Cheng J. 2023. Characterization of *Leuconostoc mesenteroides* MJM60376 as an oral probiotic and its antibiofilm activity. Mol Oral Microbiol 38:145–157. doi:10.1111/omi.12397. 36306428

[26] Kreth J, Zhang Y, Herzberg MC. 2008. Streptococcal antagonism in oral biofilms: *Streptococcus sanguinis* and *Streptococcus gordonii* interference with *Streptococcus mutans*. J Bacteriol 190:4632–4640. doi:10.1128/JB.00276-08. 18441055 PMC2446780

[27] Lozano CP, Díaz-Garrido N, Kreth J, Giacaman RA. 2019. *Streptococcus mutans* and *Streptococcus sanguinis* expression of competition-related genes, under sucrose. Caries Res 53:194–203. doi:10.1159/000490950. 30107374

[28] Cao G, Zhang M, Miao J, Li W, Wang J, Lu D, Xia J. 2015. Effects of X-ray and carbon ion beam irradiation on membrane permeability and integrity in *Saccharomyces cerevisiae* cells. J Radiat Res 56:294–304. doi:10.1093/jrr/rru114. 25599994 PMC4380059

[29] Hada M, Georgakilas AG. 2008. Formation of clustered DNA damage after high-LET irradiation: a review. J Radiat Res 49:203–210. doi:10.1269/jrr.07123. 18413977

[30] Bowen WH, Koo H. 2011. Biology of *Streptococcus mutans*-derived glucosyltransferases: role in extracellular matrix formation of cariogenic biofilms. Caries Res 45:69–86. doi:10.1159/000324598. PMC3068567 21346355

[31] Li B, Zhou X, Zhou X, Wu P, Li M, Feng M, Peng X, Ren B, Cheng L. 2017. Effects of different substrates/growth media on microbial community of saliva-derived biofilm. FEMS Microbiol Lett 364:fnx123. doi:10.1093/femsle/fnx123. 28854684

[32] Zhang K, Wang S, Zhou X, Xu HH, Weir MD, Ge Y, Li M, Wang S, Li Y, Xu X, Zheng L, Cheng L. 2015. Effect of antibacterial dental adhesive on multispecies biofilms formation. J Dent Res 94:622–629. doi:10.1177/0022034515571416. 25715378 PMC4485219

[33] Guo X, Zhang M, Gao Y, Li W, Lu D. 2018. Determining survival fractions of *Saccharomyces cerevisiae* in response to ionizing radiation in liquid culture. J Radiat Res 59:760–764. doi:10.1093/jrr/rry070. 30165406 PMC6251422

[34] Tian Y, He X, Torralba M, Yooseph S, Nelson KE, Lux R, McLean JS, Yu G, Shi W. 2010. Using DGGE profiling to develop a novel culture medium suitable for oral microbial communities. Mol Oral Microbiol 25:357–367. doi:10.1111/j.2041-1014.2010.00585.x. 20883224 PMC2951289

[35] Wang Z, Yang G, Ren B, Gao Y, Peng X, Li M, HKXu H, Han Q, Li J, Zhou X, Cheng L. 2021. Effect of antibacterial root canal sealer on persistent apical periodontitis. Antibiotics (Basel) 10:741. doi:10.3390/antibiotics10060741. 34207470 PMC8233789

[36] Liu D, Peng X, Wang S, Han Q, Li B, Zhou X, Ren B, Xu HHK, Weir MD, Li M, Zhou X, Cheng L. 2019. A novel antibacterial resin-based root canal sealer modified by Dimethylaminododecyl methacrylate. Sci Rep 9:10632. doi:10.1038/s41598-019-47032-8. 31337813 PMC6650501

[37] Grela E, Kozłowska J, Grabowiecka A. 2018. Current methodology of MTT assay in bacteria: A review. Acta Histochem 120:303–311. doi:10.1016/j.acthis.2018.03.007. 29606555

[38] Liu Y, Wang Z, Zhou Z, Ma Q, Li J, Huang J, Lei L, Zhou X, Cheng L, Zou J, Ren B. 2022. *Candida albicans* CHK1 gene regulates its cross-kingdom interactions with *Streptococcus mutans* to promote caries. Appl Microbiol Biotechnol 106:7251–7263. doi:10.1007/s00253-022-12211-7. 36195704

[39] Wang SP, Ge Y, Zhou XD, Xu HH, Weir MD, Zhang KK, Wang HH, Hannig M, Rupf S, Li Q, Cheng L. 2016. Effect of anti-biofilm glass-ionomer cement on *Streptococcus mutans* biofilms. Int J Oral Sci 8:76–83. doi:10.1038/ijos.2015.55. 27357319 PMC4932770

[40] Ma Q, Pan Y, Chen Y, Yu S, Huang J, Liu Y, Gong T, Zou J, Li Y. 2021. Acetylation of glucosyltransferases regulates *Streptococcus mutans* biofilm formation and virulence. PLoS Pathog 17:e1010134. doi:10.1371/journal.ppat.1010134. 34860858 PMC8673623

