# Supplementary material for: Heavy Ion Radiation Directly Induced the Shift of Oral Microbiota and Increased the Cariogenicity of Streptococcus mutans
Source: Microbiol Spectr. 2023 Jun 13;11(4):e01322-23. doi: 10.1128/spectrum.01322-23 (PMC10434067; doi:10.1128/spectrum.01322-23)
Supplement: Supplemental file 1 — Supplemental material. Download spectrum.01322-23-s0001.docx, DOCX file, 0.1 MB [file spectrum.01322-23-s0001.docx]

**16S rRNA sequencing**

In present study, the total DNA of saliva-derived biofilms samples were sequenced by Shanghai Majorbio Bio-pharm Technology Co., Ltd (Shanghai, China). In brief, the universal target V4–V5 regions of the 16S rRNA gene were PCR-amplified using barcoded primers 515 F (5′-GTGCCAGCMGCCGCGG-3′) and 907R (5′-CCGTCAATTCMTTTRAGTTT-3′). PCR reactions were performed in triplicate 20 μL mixture containing 4 μL of 5× FastPfu Buffer, 2 μL of 2.5 mM dNTPs, 0.8μL of each primer (5 μM), 0.4 μL of FastPfu Polymerase and 10 ng of template DNA. The amplicons were then extracted from 2% agarose gels and further purified by using the AxyPrep DNA Gel Extraction Kit (Axygen Biosciences, Union City, CA, USA) and quantified by QuantiFluor -ST (Promega, USA) according to the protocols. Purified amplicons were pooled in equimolar and paired-end sequenced (2×300) on an Illumina MiSeq platform (Illumina, San Diego, USA) according to the instruction.


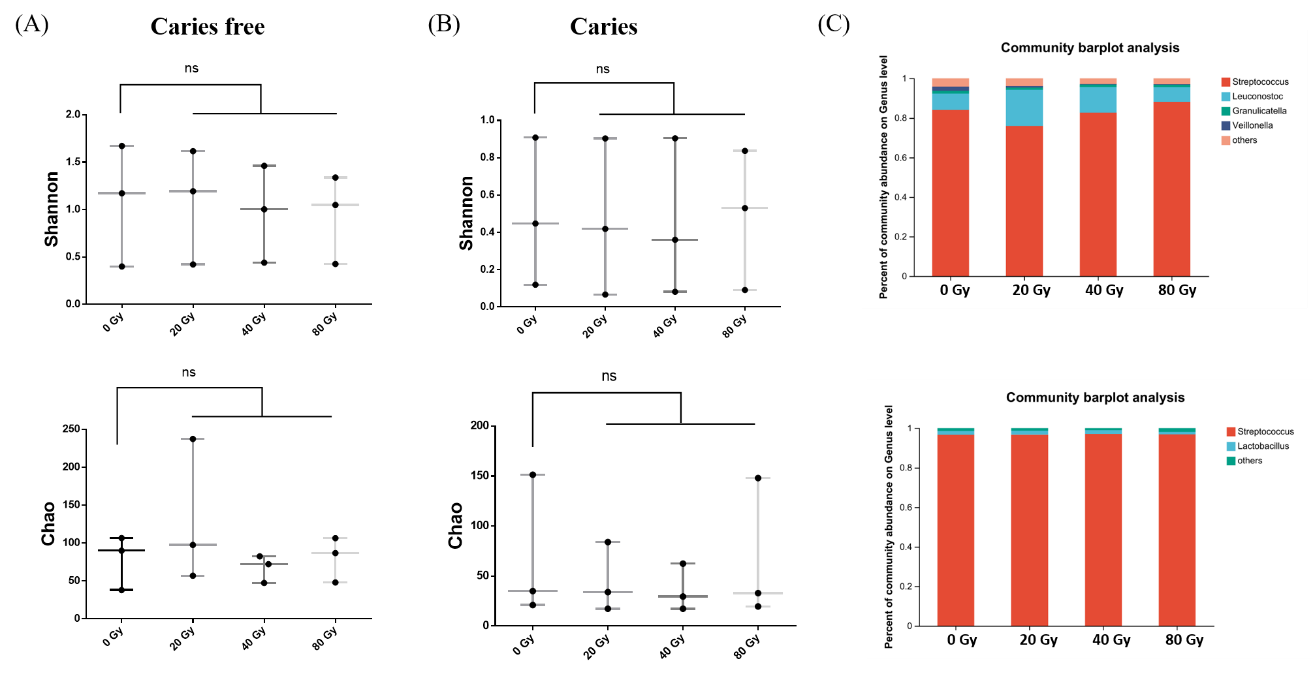


**Figure S1 16S rRNA sequencing results of oral microbial community after X-ray radiation.** (A) Shannon and Chao index of saliva-derived biofilms from caries free donors (mean ± SD; n = 3). (B) Shannon and Chao index of saliva-derived biofilms from caries donors (mean ± SD; n = 3). (C) Abundance of microorganism at the genus level of the saliva-derived biofilms. ns, not significant
